# TSCA 2.0: A New Era in Chemical Risk Management

**DOI:** 10.1289/ehp.124-A182

**Published:** 2016-10-01

**Authors:** Charles W. Schmidt

**Affiliations:** Charles W. Schmidt, MS, an award-winning science writer from Portland, ME, writes for *Scientific American*, *Science*, various *Nature* publications, and many other magazines, research journals, and websites.

A rare feat of bipartisan cooperation came to fruition on 22 June 2016 when President Barack Obama signed the Frank R. Lautenberg Chemical Safety for the 21st Century Act (HR 2576).^1^ Named for the late Senator Lautenberg (D–NJ), a lead sponsor of earlier bipartisan legislation on which the final law was based, HR 2576 reformed the Toxic Substances Control Act (TSCA),^2^ which is the major federal law regulating the safety of industrial and consumer chemicals in the United States.

**Figure:**
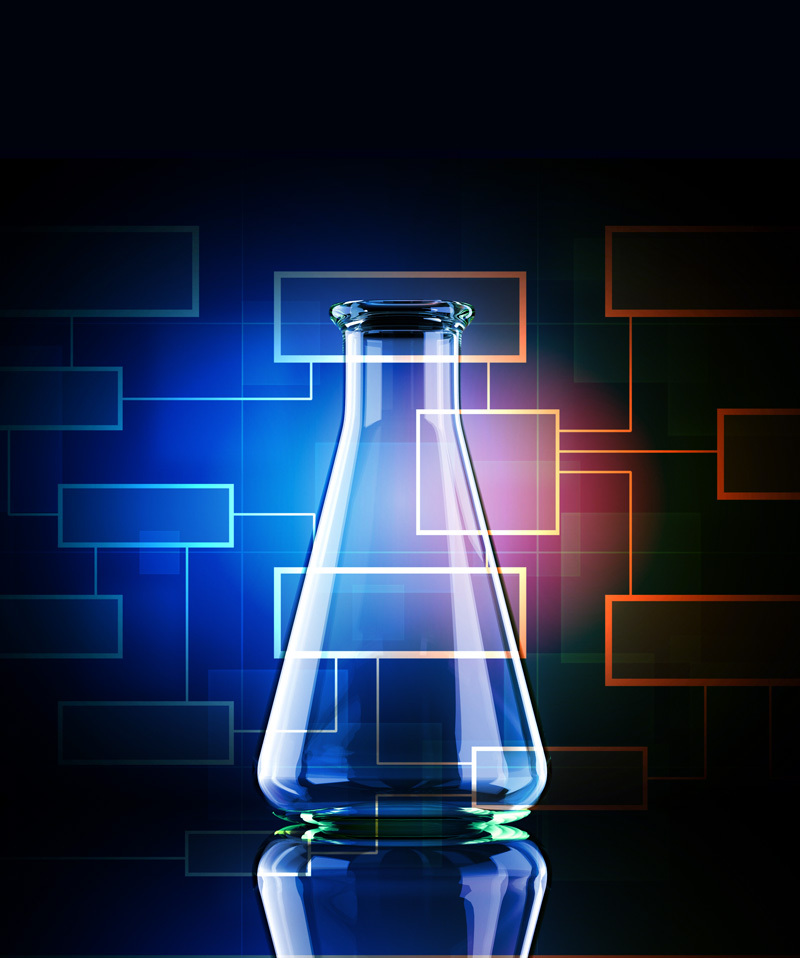
After decades of dysfunction, the Toxic Substances Control Act has been overhauled with provisions that promise better protection against potentially harmful chemicals. © Dmytro Grankin/Alamy; Alexander Aldatov/Alamy

Early indicators suggest the new law will more effectively protect people—including vulnerable populations such as children and pregnant women—than the old law it replaces. But stakeholders are watching closely to see if the changes really do live up to their promise.

## A History of Dysfunction

TSCA was passed in 1976 with the intent of giving the EPA broad powers to protect the public and the environment from potentially dangerous industrial chemicals in U.S. commerce.^3^ Importantly, TSCA’s purview did not extend to certain types or uses of chemicals that were already regulated by other laws. Pesticides, for instance, were regulated by the Federal Insecticide, Fungicide, and Rodenticide Act, while food additives, pharmaceuticals, and certain personal care products were regulated by the Federal Food, Drug, and Cosmetic Act.^4^

In passing TSCA, Congress attempted to create a regulatory system for evaluating the safety of industrial chemicals and for restricting uses when necessary to safeguard public health. Empowered by the law, EPA officials could theoretically compel companies to provide health and environmental information to fill critical data gaps. Moreover, TSCA gave them the power to regulate the manufacture, processing, distribution, use, and disposal of chemical substances found to present an “unreasonable risk” of injury to health or the environment^5^—a term the law never specifically defined.^6^

But in practice, TSCA was dysfunctional, prompting calls for reform that began decades ago. At a 2009 meeting of the Commonwealth Club of San Francisco, then–EPA administrator Lisa Jackson captured long-held sentiments by stating, “Not only has TSCA fallen behind the industry it’s supposed to regulate, it’s been proven an inadequate tool for providing the protection against chemical risks that the public rightly expects.”^7^

Two years after that meeting, Bill Chameides, then dean of the Duke Nicholas School of the Environment, blogged that under TSCA the EPA had issued regulations to control just nine of the tens of thousands of industrial chemicals in U.S. commerce. As to the rest of the chemicals, he wrote, “Well … they’re out there.”^8^

The reasons for TSCA’s failings are numerous and complex. But according to Lynn Bergeson, managing partner in the Washington, DC–based law firm Bergeson & Campbell, a key factor was that the legal hurdles the EPA had to clear before taking action under the law were excessively burdensome.

Consider what happened when the EPA tried to issue a comprehensive ban on asbestos-containing products in 1989.^9^ EPA officials were in this case motivated by evidence showing that asbestos is both a pulmonary toxicant and a known human carcinogen.^10^ But the chemical and consumer products industries were fiercely opposed to a ban, and they sued to prevent it. The resulting case—*Corrosion Proof Fittings v. EPA*—resulted in a seminal 1991 ruling against the EPA by the U.S. Court of Appeals for the Fifth Circuit, which concluded that by proposing to simply ban asbestos, the agency had failed to adequately consider less burdensome ways to reduce the risk of asbestos exposure, such as labeling products that contain it.^11^

Plaintiffs and others who felt that public exposures to asbestos were negligible hailed the decision.^12^ However, Richard Denison, a lead senior scientist with the Environmental Defense Fund (EDF), says the decision further crippled the EPA’s already hamstrung authority under the law. For the EPA to show that banning the products was the least burdensome of all potential alternatives, he says, would have required a study so exhaustive as to be virtually infeasible. The EPA never appealed the ruling, nor did it ever again attempt to issue a comprehensive ban under TSCA.

Meanwhile, roughly 62,000 chemicals already in commerce when TSCA was first enacted were for all intents and purposes exempted from the law. Charles Auer, former director of the EPA’s Office of Pollution Prevention and Toxics (OPPT), and now a consultant in Poolesville, Maryland, says that under TSCA companies had to submit available health and environmental data only when filing premanufacture notices for new chemicals. But there was no comparable policy pushing EPA the to review such data for the chemicals that were already in commerce when TSCA was enacted. “Therefore,” Auer says, “the existing chemicals were essentially grandfathered into TSCA.”

The only way the EPA could compel companies to generate new data on existing chemicals was by publishing a rule that would make it a requirement. To justify such a rule, however, the EPA first had to show that the chemicals targeted for testing could potentially present unreasonable health and environmental risks, based on the agency’s review of the existing data. But in many cases, there simply weren’t enough existing data to make that determination, putting the agency in a catch-22: wanting new data without the means to force industry to provide it, according to current OPPT director Wendy Cleland-Hamnett.

With TSCA wallowing in dysfunction, states and the marketplace stepped in to fill the regulatory void. That led to a complex patchwork of laws and voluntary programs that companies had difficulty navigating, says Mike Walls, vice president for regulatory and technical affairs at the American Chemistry Council (ACC), an industry trade group in Washington, DC.

Martha Marrapese, a partner with Washington, DC, regulatory law firm Keller and Heckman, concurs. She says, “It was very hard for companies to address ten different [state] laws if they were trying to market a product in fifty states.”

## Key Changes

According to Walls, industry frustration eventually reached the point that the ACC and its member companies began to acknowledge that TSCA reforms were urgently needed. That change in mindset, Denison adds, enabled negotiation with environmental groups, which was reflected in the first bipartisan legislation aimed at modernizing the law. Called the Chemical Safety Improvement Act,^13^ it was introduced by Lautenberg and Senator David Vitter (R–LA) just two weeks before Lautenberg died at the age of 89.

**Figure:**
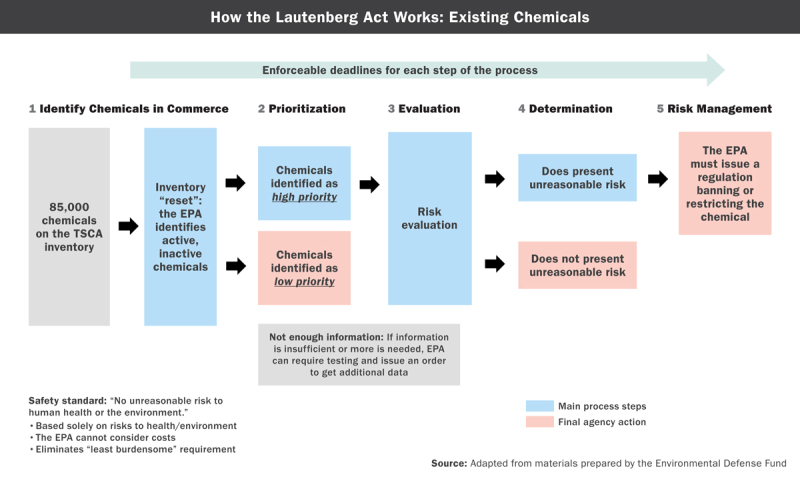
Toxic Substances Control Act (TSCA) vs. Lautenberg Act (FRL) Source: Adapted from materials prepared by the Environmental Defense Fund

Although it was widely seen as flawed,^14^ this initial legislation created a path forward on TSCA reform, Denison says, and served as a starting point for what became HR 2576. “Industry realized it needed to restore confidence in the safety of its products, and we in the health and environment community wanted to be sure we were restricting risky chemicals,” Denison says. “We had different motivations, but in TSCA reform we found common ground for dialogue.”

Some of the most important changes in the Lautenberg Act affect three core sections of the law. Section 4, which covers testing, authorizes the EPA to require companies to develop adequate test data. Section 5, which covers manufacturer notification requirements, stipulates that companies submit premanufacture notices at least 90 days prior to manufacturing, importing, or using new chemicals. It also mandates that the EPA review and make affirmative safety findings as a condition for market entry. Section 6, which covers risk evaluation and regulation, allows the EPA to restrict or ban the production, importation, or use of substances should any of those activities present unreasonable risks of health or environmental injury. It also establishes a new prioritization process and mandated review of all existing chemicals, a new safety standard that is not based on costs, and other provisions.^4^

The law specifically defines “unreasonable risk,” so that it 1) emphasizes health protection without having to consider the cost–benefit ratio of regulation, 2) focuses on whether and how chemicals are currently used, as determined by the EPA, and 3) explicitly considers and addresses the risk to subpopulations identified by the EPA as potentially exposed or susceptible.^15^ Cleland-Hamnett calls this “a very significant change.” Asked what constitutes “unreasonable risk” under the new TSCA—this being the legal threshold authorizing the EPA to take regulatory measures—she replies that officials making such a determination would rely in part on “standard benchmarks used in chemical risk assessment.” To wit: Cleland-Hamnett cites the EPA’s cancer risk benchmark of no more than 1 case out of 1 million people exposed to a given carcinogen.

Risks above that benchmark are typically deemed unacceptable by the EPA and by state health agencies.^16^ “But there isn’t any bright line demarcating ‘reasonable’ from ‘unreasonable,’” Cleland-Hamnett says. “It depends on the nature of the exposed population and the severity of the chemical’s effects. It comes down to multiple factors and the agency’s best judgment.”

Importantly, Marrapese says, the new law removes impediments to the EPA’s authority over existing chemicals. The EPA no longer has to demonstrate the potential for unreasonable risk from an existing chemical before it can require new data by rule making (although that option remains); the agency can simply order companies to generate whatever health, environmental, or exposure data it deems necessary, according to Mark Duval, a principal with Beveridge & Diamond, a Washington, DC–based law firm.

The law also mandates that the EPA determine how many of the 84,000 chemicals currently on the TSCA inventory^17^ (which includes those that were grandfathered in in 1976) are still in active use today. Like guests at the Hotel California, jokes Bergeson, chemicals on the inventory “can check in anytime they like, but they can never leave.” It’s likely that many of the chemicals on the list are no longer active, she says, but TSCA contained no mechanism for removing discontinued substances. Walls predicts the number of active chemicals at 12,000–15,000, while Denison puts the number at 30,000–50,000.

Over time, the EPA will split the active chemicals into two categories: a high-priority category for those that may present an unreasonable risk and a low-priority category for those that are deemed not to.^15^ High-priority chemicals will then undergo risk evaluations, and those with confirmed risks will proceed to risk management, which Cleland-Hamnett says means that “we will take whatever regulatory steps are needed to ensure that the unreasonable risk no longer exists.”

Auer says shortcomings under Section 4 were particularly evident during the early 1990s, after the Organisation for Economic Co-operation and Development (OECD), of which the United States is a member, set out to compile a screening information data set for high production volume (HPV) chemicals—those that are manufactured or imported at volumes in excess of 1 million pounds per year.^18^ When very little data could be uncovered for these chemicals in the U.S. market, the EDF, ACC, EPA, and other organizations launched the U.S. HPV Challenge Program^19^ to fill the gaps.

“Companies voluntarily committed to satisfying [screening information data set] requirements, and in so doing they uncovered lots of data from industry that hadn’t been previously released,” Auer says. “But then the OECD changed direction, industry support flagged, and at the end of the day, the voluntary program never fully solved EPA’s issues with inadequate test data.”

According to Walls, HPV chemicals used in the United States number roughly 3,000, and the HPV Challenge Program uncovered at least a few screening-level data for roughly 2,300 of them. In most cases, he says, those data supply basic information that the EPA can use to make additional decisions—“they’re not full-blown risk assessments,” he explains, “but enough to give the agency an idea if it needs to conduct a deeper dive.”

The new reforms, according to Auer, allow the EPA to order tiered tests for existing chemicals, starting with screening-level hazard and exposure data, and then, if warranted by screening-level findings, moving on to higher-tier tests for end points such as neurotoxicity. “I’m optimistic that through these changes, EPA can crack the test data problem and get the information it needs to conduct prioritization assessments and risk evaluations under Section 6,” Auer says. “I see this as a package that offers great promise.”

## Putting the Law into Action

Per the updated law, the EPA is required to evaluate at least 10 high-priority chemicals within six months of enactment; within three years the agency should be evaluating at least 20 chemicals at any given time, with up to three years allocated to the evaluation of any single chemical. What’s more, the costs of those evaluations (and other tasks associated with administering the new law) will be paid in part by fees levied on industry. According to Cleland-Hamnett, the act allows the EPA to collect fees amounting to 25% of the cost of performing activities under Sections 4, 5, and 6.

**Figure:**
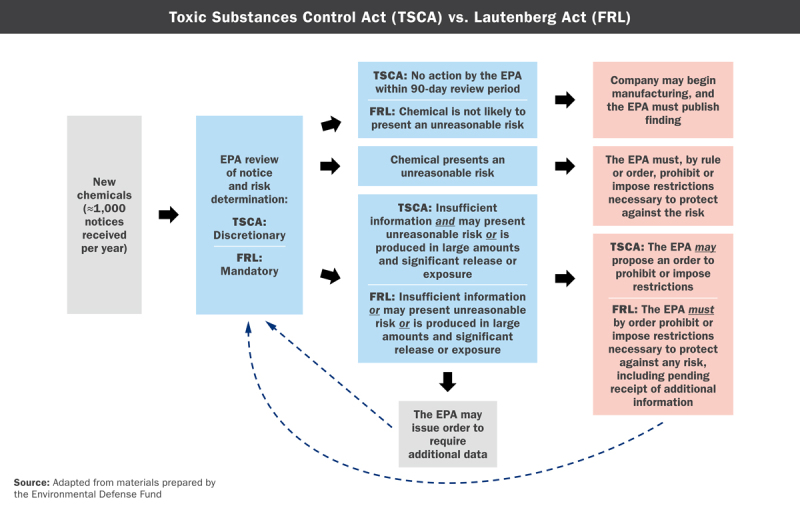
How the Lautenberg Act Works: Existing Chemicals Source: Adapted from materials prepared by the Environmental Defense Fund

The first 10 chemicals slated for evaluation, Cleland-Hamnett says, will be drawn from the TSCA Work Plan for Chemical Assessments, which was initially developed by the EPA in 2012 and updated in 2014.^20^ The plan currently covers 90 chemicals, each of them selected on the basis of the following factors: potential for causing children’s health effects; neurotoxic effects; persistence in the environment; probable or known carcinogenicity; use in children’s products or in products to which children may be highly exposed; and detection in biomonitoring programs.

The EPA has already completed evaluations of certain uses for about half a dozen of these chemicals and found significant risk from several uses, Denison says. Consistent with the new emphasis on risk reduction over balancing costs and benefits from regulation, the requirement that the EPA must select the “least burdensome” regulatory alternative—which proved insurmountable in the case of asbestos 25 years ago—has been stricken from TSCA. And that, Denison says, should make it easier for the agency to proceed with regulations slated to be proposed later this year, including restrictions on trichloroethylene in cleaning products, methylene chloride in bathtub refinishers, and *N*-methyl-2-pyrrolidone in paint strippers.

And what about that patchwork of laws that states put into place under TSCA? Federal preemption of state laws under the Lautenberg Act proved to be the thorniest issue to negotiate, say sources interviewed for this story. Some stakeholders were concerned that the reformed law could preempt stringent standards that were set by the states when TSCA proved unworkable. Washington State, for instance, banned flame-retardant chemicals that have been shown to disrupt thyroid hormones.^21^ And California’s Proposition 65, also called the Safe Drinking Water and Toxic Enforcement Act, requires the state to maintain a list of chemicals known to cause cancer or reproductive toxicity.

After debating preemption for three years, lawmakers agreed to grandfather in Proposition 65 and any other state actions taken before 22 April 2016. At the same time, the law prohibits states from enforcing statutes or laws that duplicate TSCA information requirements under Section 4, and likewise it does not allow them to ban or restrict chemical uses that the EPA has determined do not pose unreasonable risks. Unless they obtain a waiver, states therefore would be precluded from, for instance, setting more stringent standards for chemicals than those imposed by any upcoming EPA regulation.

“If EPA were to propose something less than a total ban on those chemicals, then by TSCA’s authority, the states themselves would not be allowed to,” says Daniel Rosenberg, senior attorney with the Natural Resources Defense Council. “However, states might try to ban the chemicals by invoking some other state law. So you can expect legal challenges, both from the states and also from the chemical industry.” In Rosenberg’s view, the quality of the EPA’s risk evaluations will determine if the law represents a new benefit for public health. Still, he adds, “The new TSCA is a much better law.”

Others also have praised the new TSCA. The ACC’s Walls says, “It creates a federal structure that enhances public safety while preserving an ability for companies to innovate and compete in a global market.” Denison concurs, saying, “The reformed TSCA offers significant improvements on just about every count.”

Cleland-Hamnett, whose office bears responsibility for implementing the TSCA reforms, agrees. “It’s a major accomplishment in terms of updating an environmental law that was not working well before, and a huge opportunity to protect human health and the environment,” she says. “I’m honored to have the ability to work on it.”

In August 2016 the EPA issued a call for nominations to serve on a new expert panel—the Science Advisory Committee on Chemicals—charged with providing guidance on upcoming TSCA risk assessments. The committee will consist of 14 members who meet three to four times per year. According to a *Federal Register* notice from August 26, the EPA will take nominations and comments through October 11.^22^
